# The role of vitamins and nutrients in rosacea

**DOI:** 10.1007/s00403-024-02895-4

**Published:** 2024-05-02

**Authors:** Yanci A. Algarin, Anika Pulumati, Dana Jaalouk, Jiali Tan, Keyvan Nouri

**Affiliations:** 1 Department of Dermatology, Eastern Virginia Medical School, Norfolk, VA USA; 2 Department of Dermatology, University of Missouri-Kansas City School of Medicine, Kansas City, MO USA; 3 Department of Dermatology, Albany Medical College, Albany, NY USA; 4 Department of Dermatology and Cutaneous Surgery, University of Miami Leonard M. Miller School of Medicine, Miami, FL USA

**Keywords:** B Vitamins, Omega-3 fatty acids, Rosacea, Vitamin A, Vitamin D, Zinc

## Abstract

Rosacea is a common inflammatory skin condition displaying symptoms like flushing, erythema, papules, and pustules. Oral antibiotics, despite long-term adverse effects, are often used due to topical treatment limitations, underscoring the need for cost-effective choices like dietary modifications. Our review investigates the role of vitamins and minerals in rosacea, and provides evidence-based recommendations for supplementation and topical treatment of these nutrients for rosacea. An online search was performed on PubMed, Web of Science, Science Direct, Google Scholar, and ClinicalTrials.gov from 1998 to 2023. Included studies were summarized and assessed for quality and relevance in rosacea management. Varied outcomes emerged concerning the impact of essential vitamins and minerals on rosacea treatment. Vitamin A derivatives, specifically oral isotretinoin, demonstrated significant efficacy, with a 90% reduction in lesions, complete remission in 24% of patients, and marked improvement in 57% of patients. Vitamin B3 derivatives, such as topical 1-methylnicotinamide 0.25% and NADH 1%, improved symptoms in 76.4% (26/34) and 80% of patients, respectively. Outcomes for vitamin D, vitamin C, and zinc supplementation varied across studies. However, zinc sulfate solution 5% significantly reduced acne rosacea severity for patients with 40% and 60% exhibiting a moderate or good response, respectively. Omega-3 fatty acids showed significant improvement in alleviating xerophthalmia in 64% of patients with ocular rosacea. Vitamins and minerals hold potential in managing rosacea symptoms, offering a safe and cost-effective alternative or adjunctive treatment option. Currently, there are no established recommendations regarding their supplementation for rosacea. Studies assessing serum levels of vitamins and minerals in relation to rosacea are warranted, as this avenue holds potential for future advancements in the field.

## Introduction

Rosacea is a chronic relapsing inflammatory skin condition affecting approximately 10% of the population, particularly those that are fair-skinned and of European descent [1, 2]. Clinical features include skin sensitivity, flushing, centrofacial erythema, papules and pustules [3, 4]. The symptoms of rosacea vary in severity, often exhibiting a pattern of fluctuation between exacerbation and remission, with initial stages characterized by occasional flushing. As the condition progresses, patients can develop persistent erythema, telangiectasias, and/or recurrent papules and pustules. Rosacea is divided into four main subtypes: erythematotelangiectatic, papulopustular, phymatous, and ocular [5]. Symptoms may encompass multiple subtypes simultaneously or present as isolated findings without aligning with a specific subtype.

Managing rosacea remains challenging for dermatologists. Treatment options may include gentle skin care, systemic or topical therapies, laser- and light-based therapies, invasive methods (e.g., microneedling), or combinations of these options [6]. In 2020, topical 1.5% minocycline (FMX103) was FDA approved as a suitable option for the treatment for moderate-to-severe papulopustular rosacea [7, 8]. For patients unresponsive to topical medications, oral anti-inflammatory antibiotics, specifically tetracyclines, are the mainstay of treatment. However, long-term oral antibiotic therapy raises concerns due to the potential adverse effects and risk of bacterial resistance. Therefore, exploring alternative or adjunctive treatments for rosacea, such as vitamins and minerals, have received renewed interest. Vitamins and minerals can have various effects on skin health and inflammation, and understanding their impact on rosacea symptoms could provide valuable insights for medical professionals and patients.

Recent literature increasingly examines the impact of vitamins and minerals on rosacea, emphasizing their potential as adjunctive therapies [9, 10]. Studies have focused on topical and oral forms of key vitamins and minerals like vitamin A, vitamin D, zinc, and omega-3 fatty acids (ω-3 FAs) [9, 11]. Exploring the potential benefits of these essential nutrients for managing rosacea symptoms presents an encouraging avenue of research. It allows us to identify potential patterns of deficiency or imbalance in these vitamins and minerals among rosacea patients, guiding critical areas for further investigation. Optimizing the use of these micronutrients through topical or oral supplementation has the potential to reduce flare-ups, promote lesion resolution, and enhance the overall quality of life for individuals with rosacea.

The primary aim of this review was to assess the vitamins and minerals that have been proven beneficial in managing rosacea, either orally or topically. Our focus also extends to examining the patterns of vitamin and mineral imbalances commonly observed in rosacea patients. Relevant studies will be summarized and graded for quality of evidence (Table 1). Evidence-based recommendations concerning the use of these vitamins and minerals, whether through oral supplements or topical administration, in the management of rosacea will be made, providing clinicians a framework by which to recommend or dissuade certain interventions for patients.

**Table 1:** Grades of recommendation [12]

Grade of recommendations	Level of evidence	Type of study
A	1a	Systematic review of (homogeneous) randomized controlled trials
A	1b	Individual randomized controlled trials (with narrow confidence intervals)
B	2a	Systematic review of (homogeneous) cohort studies of “exposed” and “unexposed” subjects
B	2b	Individual cohort study/low-quality randomized control studies
B	3a	Systematic review of (homogeneous) case–control studies
B	3b	Individual case–control studies
C	4	Case series, low-quality cohort or case–control studies
D	5	Expert opinions based on non-systematic reviews of results or mechanistic studies

## Methods and study design

### Search

We performed a comprehensive search strategy via PubMed, Web of Science, Science Direct, Google Scholar, and ClinicalTrials.gov from 1998 to 2023 by using the following search terms and keywords: “rosacea,” OR “acne rosacea,” OR “ocular rosacea,” AND one of the following search terms: “vitamin A,” OR “vitamin C,” OR “vitamin D,” OR “vitamin E,” OR “vitamin K,” OR “thiamin,” OR “vitamin B1,” OR “riboflavin,” OR “vitamin B2,” OR “niacin,” OR “vitamin B3,” OR “pantothenic acid,” OR “vitamin B5,” OR “pyridoxine,” OR “vitamin B6,” OR “biotin,” OR “vitamin B7,” OR “folate,” OR “vitamin B9,” OR “cobalamin,” OR “vitamin B12,” OR “calcium,” OR “phosphorus,” OR “potassium,” OR “sodium,” OR “chloride,” OR “magnesium,” OR “iron,” OR “zinc,” OR “iodine,” OR “sulfur,” OR “cobalt,” OR “copper,” OR “fluoride,” OR “manganese,” OR “selenium.” Only articles published in English were included. Once articles were deemed relevant to the research study, they were summarized, and assessed for biases and recommendations. References from relevant articles were also used to locate more articles for use as support for our study. The six vitamins and minerals most frequently found to play a role in rosacea management include vitamin A, vitamin B3, vitamin B12, vitamin D, Vitamin K, zinc and ω-3 FAs, which are the focus of this review.

### Inclusion criteria

The inclusion criteria of our study were articles comprising randomized clinical trials, systematic reviews, meta-analyses, well-designed controlled trials, and prospective comparative cohort trials. Case–control and retrospective cohort studies, case series, and case reports were also included if they evaluated the role of vitamins and nutrients in patients with rosacea, and the information was not available in reviews or trials. Articles lacking sufficient information or duplicate publications were excluded.

## Rosacea and the key vitamins and minerals

### Vitamin A

Vitamin A, a fat-soluble micronutrient, influences various physiological and immunological processes within the body primarily through two active metabolites: retinoic acid and retinol. Limited studies explore the use of vitamin A or its derivatives for rosacea, but some studies suggest potential benefits. In addition to their immunomodulatory behavior and subsequent anti-inflammatory effects, vitamin A derivatives play a crucial role in regulating keratinocyte proliferation and differentiation, thereby increasing the turnover of the follicular epithelium [13]. These characteristics offer a plausible explanation for their potential in alleviating erythema and inflammation in individuals with rosacea.

Many studies have assessed the use of topical and oral retinoids for rosacea treatment [14–19]. The specific mechanisms by which these derivatives affect rosacea remain unclear, but they appear promising in reducing inflammatory lesions and improving skin texture. A double-randomized control trial (RCT) by Chang et al. evaluated a clindamycin phosphate 1.2% and tretinoin 0.025% gel in 79 patients with moderate to severe papulopustular rosacea over 12 weeks [20]. While no significant differences in papule or pustule counts were observed between the placebo and treatment groups, the combination gel notably improved the telangiectatic component and effectively managed the erythematotelangiectatic subtype in the treatment group. However, attributing these benefits to clindamycin or tretinoin independently remains challenging [20].

Adapalene, a synthetic retinoid, affects cellular differentiation, keratinization, and inflammation, suggesting its potential use in treating rosacea. In a study by Altinyazar et al., 55 papulopustular rosacea (PPR) patients were randomized to receive adapalene gel 0.1% or metronidazole gel 0.75% over 12 weeks [21]. The adapalene group showed a significant reduction in inflammatory lesions compared to the metronidazole group. However, only the metronidazole group demonstrated a significant reduction in erythema and telangiectasias. These results show that adapalene gel could be an effective alternative or adjunct treatment for PPR, particularly in addressing inflammatory lesions. The study underscores the complexity of rosacea treatment, emphasizing the need for a combination of therapies for optimal management [21].

Oral isotretinoin, used off-label for rosacea since the 1980s, has shown positive outcomes in refractory cases. A multicenter trial involving 92 patients with severe PPR reported significant improvement following a 20-week isotretinoin regimen [22]. Another study of 22 patients with recalcitrant PPR observed a decrease in inflammatory lesions and erythema within nine weeks on a daily 10 mg dose, further improving by week 16 [22]. Gollnick et al.'s double-blind RCT highlighted isotretinoin's efficacy in PPR and phymatous rosacea, noting a 90% lesion reduction and higher complete remission rates than doxycycline [23]. These findings emphasize the utility of vitamin A derivatives in managing specific rosacea subtypes. Continuous microdose isotretinoin has been suggested as an alternative for challenging, recalcitrant rosacea cases, potentially reducing relapse rates post-discontinuation. Proposed regimens include initial 10–20 mg/day doses for 4–6 months, followed by 0.03 to 0.17 mg/kg/day maintenance microdoses for up to 33 months.[24, 25]. Despite known side effects, which are predictable and manageable, this approach presents a viable option over repetitive conventional oral antibiotic treatments. Overall, oral isotretinoin stands out as one of the few treatment options demonstrating efficacy across various rosacea subtypes, particularly in PPR, erythematotelangiectatic rosacea, and phymatous rosacea.

Vitamin A derivatives in rosacea treatment have been well-established. These derivatives exhibit potent anti-inflammatory and sebum-regulating properties that help to manage the underlying factors contributing to the progression of rosacea. Unlike pure vitamin A supplementation, which carry risks of toxicity and adverse effects, these derivatives are formulated to harness the therapeutic benefits of vitamin A while minimizing potential drawbacks. Thus, physicians can confidently leverage these derivatives for targeted rosacea treatment.

### B vitamins

Few studies evaluating the status of B vitamins in rosacea have been published. Limited studies suggest that deficiency in vitamins B2, B9, and B12 are linked to rosacea [26–28]. Most recently, Chung et al. found that PPR severity correlated significantly with decreased levels of B12 and B9. There was also a positive correlation between the severity of PPR and serum Hcy levels [27]. Studies have shown that specific B vitamins, notably B3 and B12, may influence rosacea symptoms, exhibiting both beneficial and exacerbating effects [27–35].

Vitamin B3, existing as niacin and nicotinamide, are chemically similar yet functionally distinct. Both support various physiological functions, with niacin being convertible to niacinamide in the body [36, 37]. Despite their similar biological roles, they differ pharmacologically: niacin is a lipid-lowering agent known for inducing flushing [29, 30]. While nicotinamide offers benefits such as improving skin barrier functions and possesses anti-inflammatory properties, potentially mitigating the redness and inflammation characteristic of rosacea [31, 32]. In a pilot study by Wozniacka et al., 34 rosacea patients were treated for 2 weeks with a topical gel containing 0.25% of 1-methylnicotinamide (MNA), a metabolite of nicotinamide. The study found that 76.4% (26/34) of patients experienced improvements, suggesting that MNA may be a potential treatment option for rosacea [33]. Another clinical study evaluated the efficacy of topical 1% NADH in 10 patients with rosacea. The degree of erythema, telangiectasias, papules and pustules in each patient was evaluated before and after 2 weeks of treatment. Marked reduction in erythema and pustules were observed in 30% (3/10) of patients and moderate improvement was observed in 50% (5/10) of patients. The study noted that no side effects were observed, indicating that NADH may be a safe alternative treatment for rosacea [34].

Vitamin B12 (cobalamin) is found naturally in food and is only synthesized in bacteria [37]. It acts as a cofactor in cellular methylations [38]. Earlier research suggested that high doses of B12 and B6 could trigger rosacea fulminans, a rare and severe form of rosacea [35]. A case series study by Huang et al. examined the efficacy of hydroxocobalamin in treating rosacea. Hydroxocobalamin is a naturally occurring form of vitamin B12 that is often used intramuscularly [39]. It has a longer half-life and has been used to treat a range of medical conditions [40, 41] In the study, 13 rosacea patients received 1–4 weekly intramuscular injections of hydroxocobalamin. Skin surface temperature (SST) was evaluated using the Clinician’s Erythema Assessment (CEA) by photography and an infrared thermometer. The study concluded that there was a significant decrease in the CEA and STT levels [27]. One potential drawback of this study is its lack of randomization and control, which could introduce bias. Additional research studies are warranted to gain a comprehensive understanding of its efficacy and long-term therapeutic effects.

No RCT evaluating adjunctive B vitamin supplementation in rosacea patients exists. Only small pilot studies and case series have demonstrated that topical applications of 1-MNA and NADH are effective in improving rosacea symptoms. Although these findings underscore the potential of B vitamins as therapeutic agents in rosacea treatment, larger, more comprehensive, and rigorously controlled studies are needed to fully elucidate the efficacy and safety of these treatments in the long term. Additionally, while the study by Chung et al. breaks new ground by being the first to analyze serum levels of homocysteine, vitamin B12, and folic acid in individuals with rosacea, it also uncovers an area that warrants further exploration. Specifically, the unexplored potential of B9 and B12 supplementation as preventive therapy for rosacea.

### Vitamin D

Vitamin D, or calciferol, is a vital fat-soluble hormone derived from dietary sources and skin production upon UV-B ray exposure. Its active form, 1.25-dihydroxyvitamin D (25OHD), is crucial for maintaining calcium and phosphorus balance and promoting bone health. Importantly, vitamin D3 plays a role in regulating the immune system, affecting both innate and adaptive immune responses [42]. However, the evidence regarding the relationship between Vitamin D status and rosacea is limited and inconsistent. Park et al. has shown that rosacea patients have significantly lower serum vitamin D levels (12.18 ± 5.65 ng/ml) than healthy controls (17.41 ± 6.75 ng/ml) [43]. However, other cross-sectional studies have shown that vitamin D levels are significantly higher in rosacea patients, suggesting that increased vitamin D levels may lead to rosacea [42, 44–46]. One study suggests that vitamin D levels can be used for diagnosis of rosacea at a cut off point of 19.6 with 80% sensitivity, 75% specificity, and 78% accuracy [47]. It is important to acknowledge that each of these studies utilized a cross-sectional approach, were limited by small sample sizes, and did not consider potential confounding variables like physical activity levels, amount of sun exposure, and sleep patterns. Recently, a large prospective study assessed serum 25OHD levels on the risk of incident rosacea, and found a significant association between a higher serum 25OHD levels and a decreased risk of incident rosacea [48]. Additionally, the study revealed a direct dose–response correlation between serum 25OHD levels and the occurrence of rosacea, with higher 25OHD concentrations showing a more pronounced protective effect against rosacea, particularly in young male participants.

Ultimately, the exact causative relationship between vitamin D and rosacea is yet to be determined. Currently, there are no studies that have evaluated vitamin D supplementation or topical vitamin D as a management strategy for rosacea. Further in-depth research through large, well-structured RCT is necessary to fully understand the potential therapeutic benefits of these observations. While there is some indication that vitamin D might play a role in the development of rosacea, current evidence is insufficient to determine whether vitamin D supplementation can either alleviate or worsen its symptoms.

### Vitamin K

Vitamin K, when applied topically to the skin, serves various therapeutic roles. It has been shown to be effective in mitigating aging-related vascular symptoms, wound healing, and reducing skin pigmentation issues such as periorbital hyperpigmentation. Additionally, it is beneficial in the resolution of bruising and the treatment of superficial vascular conditions like facial telangiectasia, especially when used in conjunction with other topical vitamins [49–51]. However, research on the role of vitamin K in rosacea is scarce. One recent RCT assessed the effectiveness of topical vitamin K cream 1% in treatment of steroid-induced rosacea (SIR) in 75 female patients [52]. These patients, all diagnosed with SIR, were divided into two groups with one group receiving placebo (n = 25), and the other group treated with 1% topical vitamin K cream daily for 8 weeks (n = 50). The results indicated a significant reduction in the mean erythematotelangiectasia severity score in the topical vitamin K group, decreasing from 7.5 initially to 4.24 by week 4 and further to 2.6 by week 8 (P = 0.013). While the mean SIR score decreased in the placebo group, it was not statistically significant. It was concluded that vitamin K can be an effective therapeutic option for treating SIR, demonstrating rapid improvement in symptoms such as flushing, persistent erythema, telangiectasia, burning sensations, and dryness, while exhibiting only mild and tolerable side effects [52]. Although these results are promising, this has been the only study to assess topical vitamin K in rosacea. More studies with larger patient samples are needed to validate these results, and to determine its true efficacy in rosacea.

### Zinc

Zinc is fundamental for the development of the cell-mediated innate immune system and functions as an antioxidant and anti-inflammatory molecule [53]. Limited trials have evaluated the use of zinc in the management of rosacea, and of the available studies, findings have been inconsistent.

Recent studies have explored the therapeutic potential of zinc in rosacea management. Sharquie et al. reported that oral zinc sulfate, administered at a dose of 100 mg thrice daily for three months (n = 25), led to a significant reduction in papules and pustules, an effect that persisted during a subsequent three-month placebo phase, suggesting both treatment and prophylactic benefits [54]. However, these findings were not replicated in a study by Bamford et al. (n = 44), where oral zinc therapy did not demonstrate a significant advantage over placebo in improving rosacea severity over 90 days. Notably, the effectiveness of zinc supplementation was found to be lower than that of the placebo, resulting in the premature termination of the study [55]. The inconsistency in outcomes may be attributed to unmeasured baseline serum zinc levels, raising questions about the differential benefits based on existing zinc status. Currently, it is unclear whether zinc supplementation is helpful for all patients or only for those with low serum zinc levels.

Topically, Sharquie et al. found a 5% zinc sulfate solution (n = 10) to be significantly more effective than a 2% tea lotion (n = 8) in reducing the severity of acne rosacea over two months (p = 0.00003) [56]. The zinc solution showed a moderate response in 40% of cases and a good response in 60% of patients, with no serious adverse effects. Although the 2% tea lotion showed some benefits, it did not achieve a statistically significant level of efficacy compared to zinc sulfate (P = 0.1). This suggests that zinc sulfate solution is a simple, effective, and safe option as a topical therapy or in combination with oral therapy.

Despite some positive findings, the efficacy of oral zinc in rosacea remains debatable. Conversely, topical zinc sulphate, noted for its simplicity, safety, and cost-effectiveness, appears to be a promising monotherapy or adjunct for rosacea treatment [56]. Limitations of these studies include the small cohort size, which was predominantly Caucasian, potentially affecting the generalizability of the findings. More studies with larger sample sizes are needed to determine the benefits for zinc monitoring and supplementation in rosacea management.

### Omega–3-fatty acids

Omega-3 fatty acids (FAs), including eicosapentaenoic acid (EPA) and docosahexaenoic acid (DHA), have shown promise in managing ocular symptoms of rosacea. Ocular rosacea is characterized by a myriad of symptoms, among which itchy and dry eyes are common complaints [57, 58]. In a RCT involving 518 patients with dry eye symptoms, including those with ocular rosacea, supplementation with 325 mg of EPA and 175 mg of DHA twice daily for three months resulted in 65% of the omega-3 group showing significant improvement, compared to 33% in the placebo group. (P = 0.005) [59]. Another RCT by Bhargava et al. evaluating rosacea patients (n = 130) with dry eye symptoms confirmed these findings, where omega-3 FA capsules led to a significant relief in symptoms over six months over placebo, without any serious side effects (p < 0.001) [60]. These consistent outcomes suggest that omega-3 FAs could be beneficial in the treatment of dry eye symptoms associated with rosacea, although the necessity of supplementation regardless of baseline omega-3 FA levels requires further investigation.

Recently, Shen et al. examined the benefits of omega-3 FAs for rosacea treatment using both in vivo and in vitro approaches [61]. Their study showed that a diet high in omega-3 s helped reduce symptoms such as erythema and inflammation in rosacea-like conditions in mice by exerting inhibitory effects on inflammatory and immune responses, and also angiogenesis. These experimental findings further support omega-3 supplements as a promising and safe treatment for rosacea, though it is not confirmed if a deficiency in omega-3 FAs contribute to the condition [61]. Further clinical trials are warranted to determine the effectiveness of omega-3 FAs in rosacea management. Table 2 outlines supplementation and treatment recommendations for the vitamins and minerals used in the management of rosacea. Table 3 provides a summary of the articles reviewed in our study, highlighting key findings.

**Table 2:** Supplementation and topical treatment recommendations for Rosacea

Vitamin or mineral	Recommendation	Grade and level of evidence
Vitamin A	Oral isotretinoin is safe and can be recommended for recalcitrant cases of rosacea and papulopustular rosacea No recommendations to measure or monitor serum vitamin A levels	Grade A, level 1b
	Insufficient evidence to recommend topical adapalene for rosacea	Grade B, level 2b
Vitamin B3	Insufficient evidence for supplementation recommendation No recommendations to measure or monitor serum vitamin B3 levels	Grade D, level 5
	Topical 1-methylnicotinamide is a safe alternative to conventional treatment for rosacea, and can be recommended	Grade D, level 5
	Topical NADH is a safe alternative to conventional treatment for rosacea, and can be recommended	Grade D, level 5
Vitamin B12	Insufficient evidence for supplementation recommendation No recommendations to measure or monitor serum vitamin B12 levels	Grade D, level 5
	Intramuscular B12 (hydroxycobalmin) is a safe alternative to conventional treatment and can be used to treat persistent rosacea	Grade D, level 5
Vitamin D	Insufficient evidence for supplementation recommendation No recommendations to measure or monitor serum vitamin D levels	Grade D, level 5y
Vitamin K	Insufficient evidence for supplementation recommendation No recommendations to measure or monitor serum vitamin K levels	Grade B, level 2b
	Topical Vitamin K is safe and can be recommended for steroid-induced rosacea, however standard treatment approaches should be attempted first	Grade B, level 2b
Omega-3 FAs	Supplementation is safe and recommended for ocular rosacea patients or rosacea patients with dry eye symptoms No recommendations to measure or monitor serum omega-3 FAs levels	Grade B, level 2b
Zinc	Insufficient evidence for supplementation recommendation No recommendations to measure or monitor serum zinc levels	Grade B, level 2b
	Topical zinc sulfate is safe and can be recommended for rosacea in mild cases and could be combined with other standard oral therapies such as azithromycin, doxycycline, and others	Grade B, level 2b

*NADH* Nicotinamide Adenine Dinucleotide (Hydrogen), *FAs* Fatty acids

**Table 3:** Summary of key findings for vitamins and minerals in managing Rosacea

Study Authors	Study Design	Year	Country	Patient number (N)	Follow-up	Data collected	Key Findings
Vitamin A							
Chang et al.	Pilot study, RCT	2012	USA	79	12 weeks	• Statistically significant reduction in absolute papule or pustule count after 12 weeks of usage (combination topical clindamycin phosphate 1.2% and tretinoin 0.025%)	• No significant difference in lesion count between placebo and treated groups after 12 weeks (P = 0.10) • Near significant improvement in telangiectasias (P = 0.06) and in erythematotelangiectatic rosacea subtype (P = 0.05) in the treated group after 12 weeks
Altinyazar et al.	RCT	2005	Turkey	55	12 weeks	• Used topical adapalene gel (0.1%) and topical metronidazole gel (0.75%) • Inflammatory papules, pustules, erythema, and telangiectasias were assessed at baseline and at 2, 4, 8, and 12 weeks. Side effects were noted during each visit	• Adapalene group had significantly fewer inflammatory lesions than metronidazole group • No significant difference in erythema and telangiectasia scores in the adapalene group; significant reduction in erythema in metronidazole group
Gollnick et al.	RCT	2010	Germany	573	N/A	• Assessed the effectiveness of systemic isotretinoin in the treatment of severe forms of rosacea, specifically subtype II and III	• 0.3 mg/kg isotretinoin significantly outperformed placebo and was significantly non-inferior to doxycycline (90% vs. 83% reduction in lesions) • Isotretinoin: 24% complete remission and 57% marked improvement versus Doxycycline: 14% complete remission and 55% marked improvement • 0.5 mg/kg isotretinoin resulted in increased dermatitis facialis compared to 0.3 mg/kg dose
Hofer	Observational cross-sectional study	2003	Switzerland	12	N/A	• Assessed the psychological benefits for patients receiving CMI via the DLQI	• No symptomatic side effects and normal blood test values in patients on CMI maintenance therapy at 0.03–0.17 mg/kg daily • Mean DLQI score in the CMI group was low at 1.16; the untreated group had a higher mean DLQI score of 8.1
Vitamin B3							
Wozniacka et al.	Pilot study	2005	Poland	34	7 months	• Statistical analysis of the number of papules and pustules, erythema, and patient complaints after 1, 2, 3, and 4 weeks of topical 0.25% MNA twice daily treatment	• Clinical efficacy was seen after 1 week of treatment • Statistically significant improvement (p < 0.001) was observed in the number of papules and pustules, erythema, and patient complaints
Wozniacka et al.	Pilot study	2003	Poland	10	4 weeks	• NADH in a hydrophobic base was applied on patients twice daily. Erythema, papules, and pruritus were observed	• Significant improvement (75% reduction) in papules and erythema was observed in 3 patients. Moderate improvement (50% reduction) was observed in 5 other patients. Slight improvement in irritation and erythema was observed in one patient
Vitamin B12							
Huang et al.	Case Series	2022	Taiwan	13	1–4 months	• CEA by photography and infrared thermometer to evaluate SST before and after 1 to 4 weekly intramuscular injections of hydroxocobalamin	• 92% (12/13) patients had significant improvement within one hour of injection • CEA was reduced from 2.2 ± 0.6 to 1.2 ± 0.4 (p < 0.001). SST from both cheeks was significantly reduced from 36.7 ± 0.7℃ to 36.2 ± 0.61℃ (p < 0.001)
Chung et al.	Case–control	2022	Korea	196	N/A	• Serum levels of vitamin B12, vitamin B9, Hcy were measured • A correlation was assessed between PPR severity and serum levels of B12, B9 and Hcy	• Serum B12 and B9 levels were significantly lower in PPR group (p = 0.011) than controls (p = 0.0173) • PPR severity was positively correlated with serum Hcy levels (p < 0.001)
Vitamin D							
Ekiz et al.	Case–control	2014	Turkey	44	N/A	• Serum levels of 25-hydroxyvitamin D, calcium, and intact parathyroid hormone	• Higher average vitamin D level in rosacea group vs controls (21.4 ± 9.9 vs 17.1 ± 7.9 ng/ml, p = 0.04) • 38.6% of rosacea patients were vitamin D deficient, compared to 28.1% of controls (p = 0.34) • No significant differences in calcium and PTH levels between groups (p = 0.21, p = 0.49)
Hagag et al.	Prospective case–control	2021	Egypt	30	N/A	• Serum levels of 25-hydroxyvitamin D, total calcium, and ionized calcium • Comprehensive dermatological examination, which included evaluating rosacea distribution, clinical variants, extent, and disease severity using a rosacea clinical scoring system	• Rosacea group showed significantly higher average vitamin D levels vs. controls, (25.5 ± 5.3 vs. 17.7 ± 5.2 ng/ml, p < 0.001) • There was a significant difference in vitamin D status between patients and controls (p = 0.010), but no significant difference in total and ionized calcium levels (p = 0.662, 0.888) • Moderate flushing and non-transient erythema were significantly associated with optimal vitamin D levels (p = 0.020 and p = 0.030, respectively)
Park et al.	Case–control	2018	Korea	34 patients for serum samples 38 patients for tissue samples	N/A	• Blood samples to test serum 25-hydroxy-vitamin D and cathelicidin levels • Tissue samples to assess cathelicidin and vitamin D receptor expression levels using the IID	• Lower average vitamin D level in rosacea group vs controls (12.18 ± 5.65 vs. 17.41 ± 6.75 ng/ml, p = 0.001) • Elevated serum cathelicidin in rosacea group (85.0 ± 26.1 ng/ml) vs. controls (55.0 ± 23.3 ng/ml, p = 0.001) • Higher average cathelicidin expression in rosacea group vs. controls (5.21 vs.4.03, p = 0.045) • No significant difference in vitamin D receptor expression between rosacea group (5.13 ± 2.4) and controls (5.03 ± 2.1, p = 0.936)
Akdogan et al.	Case–control	2018	Turkey	120	N/A	• Serum 25OHD_3_ levels and five VDR gene SNPs (Cdx2, FokI, ApaI, BsmI and TaqI) and compared between patients and HCs	• Serum 25OHD_3_ levels were higher in rosacea patients (12.9 ± 6.8) compared to controls (10.5 ± 3.7, p < 0.001) • Patients with high levels of Serum 25OHD_3_ had a 1.36-fold increased rosacea risk (95% CI 1.17–1.58) • ApaI Polymorphisms: Heterozygous: 5.26 × increase in rosacea risk (95% CI 1.51–18.35) Mutant: 3.69 × increase (95% CI 1.19–11.48) • TaqI Polymorphisms: Mutant: 4.69 × decrease in risk (95% CI 1.37–16.67) • Cdx2 Alleles: Heterozygosity: Increases rosacea risk • Wild-Type ApaI and Mutant TaqI alleles: Decrease rosacea risk
Gürel et al.	Case–control	2018	Turkey	100	N/A	• Serum vitamin D levels, calcium levels, and PTH levels were measured and compared	• Vitamin D levels were significantly higher in rosacea patients (10.55 ng/ml) than control groups (8.50 ng/ml) • Calcium levels were significantly higher in controls (8.55 mg/dl) compared to patients with rosacea (8.20 mg/dl)
Mao et al.	Cross sectional cohort study	2023	United Kingdom	370,209	13.22 years (average)	• Assess the association of serum 25OHD concentrations and VDR polymorphisms with the risk of incident rosacea	• Increased levels of serum 25OHD were inversely correlated with the risk of incident rosacea • A significant association, with each SD increase in serum 25OHD concentrations correlating to a 23% reduced risk of rosacea (HR = 0.77, 95% CI: 0.63, 0.93) • Patients with vitamin D (25OHD) levels above 50 nmol/L had a 19% lower risk (HR: 0.81, 95% CI: 0.70, 0.94) of developing rosacea compared to those with levels below 25 nmol/L • Patients with 25OHD levels over 75 nmol/L and the TaqI GG allele showed a 49% reduced risk (HR: 0.51, 95% CI: 0.32, 0.81) compared to individuals with 25OHD under 25 nmol/L and the TaqI AA allele
Vitamin K							
Abdullah et al.	RCT, single blind	2020	Iraq	75	8 weeks	• Erythematotelangiectatic severity scoring system after topical vitamin K cream 1%	• Significant reduction in rosacea severity score for vitamin K group decreasing from 7.5 to 4.24 (week 4) and further to 2.6 (week 8) (p = 0.013) • Placebo decreased from 7.76 to 6.64 (week 4) and further to 6.4 (week 8), and was not statistically significant (p = 0.185)
Zinc							
Sharquie et al.	RCT, double blind	2012	Iraq	25	6 months	• Rosacea severity score after oral zinc sulphate (100 mg, 3 times a day)	• Significant improvements in rosacea severity score (p < 0.01)
Bamford et al.	Randomized double-blind trial	2006	USA	44	3 months	• Improvement in rosacea with zinc sulphate (220 mg, 2 times a day)	• Both groups had an improvement in their rosacea and there was no difference between groups
Sharquie et al.	Single-blinded comparative therapeutic clinical trial,	2014	Iraq	22	2 months	• Clinical improvement was evaluated every 2 weeks by determination of the disease severity score (Sharquie’s score) before and after treatment. (5% Topical zinc sulfate solution vs. 2% tea lotion)	• Five percent zinc sulfate solution was statistically significant in reducing the disease severity score in acne rosacea. (p = 0.00003)
ω-3 FAs							
Bhargava et al.	RCT, double blind	2013	India	518	3 months	• Comprehensive assessments during each visit included CDVA, slit lamp exams, and a graded dry eye symptom questionnaire (mild, moderate, severe) • Treatment response was evaluated using standard tear function tests: Schirmer I, TBUT, Rose Bengal staining, and conjunctival impression cytology	• There was a significant change in both Schirmer's test value and TBUT values in the omega-3 group (P < 0.001), both comparisons • The mean reduction in symptom score in omega-3 group was 2.02 ± 0.96 as compared to 0.48 ± 0.22 in placebo group (P < 0.001)
Bhargava et al.	RCT, double blind	2016	India	130	6 months	• Subjective dry eye symptoms and in Meibomian gland score, Schirmer score and tear film breakup time (measures of eye dryness) • Groups were randomized to receive omega-3 fatty acids (180 mg EPA and 120 mg DHA) or a placebo twice daily	• There was a significant change in subjective dry eye symptoms and in Meibomian gland score, Schirmer score and TBUT. (p < 0.001)

*25-hydroxyvitamin D3* 25OHD_3_, *CMI* Continuous microdose isotretinoin, *DLQI* Dermatology Life Quality Index, *CEA* Clinician’s Erythema Assessment, *SST* Skin surface temperature, *CDVA* Corrected distance visual acuity, *TBUT* Tear break-up time, *IID* immunostaining-intensity-distribution index, *PPR* papulopustular rosacea, *CI* Confidence interval, *Hcy* Homocysteine, *SNPs* single nucleotide polymorphisms, *PTH* parathyroid hormone, *VDR* Vitamin D receptor, *SD* Standard deviation

## Conclusion

Rosacea is a complex skin condition with various clinical subtypes and manifestations, making its management challenging for dermatologists. Vitamins and minerals have the potential to manage rosacea symptoms and offer a safe and cost-effective alternative or adjunctive treatment option. Vitamin A derivatives, particularly oral isotretinoin, have been shown to be effective in treating various subtypes of rosacea, and vitamins B3 and B12 may offer anti-inflammatory benefits to rosacea. Additionally, topical vitamin K shows promise in reducing severity of SIR. The relationship between vitamin D levels and rosacea remains inconclusive, necessitating further research to clarify its therapeutic role. Similarly, the efficacy of oral zinc supplementation in rosacea treatment is still under debate, but topical zinc shows significant promise. Omega-3 fatty acids have emerged as a potential treatment for ocular symptoms associated with rosacea, demonstrating significant improvement in RCT.

Currently, there are no established recommendations regarding supplementation or treatment with vitamins and minerals for rosacea. This review aims to fill this gap, providing evidence-based recommendations regarding supplementation and topical treatment for rosacea with the vitamins and minerals discussed. The current scarcity of data on the serum levels of vitamins and minerals in relation to rosacea highlights the need for further research in this area. Studies with larger sample sizes and more RCT are also needed to provide a more comprehensive understanding of the role of these nutrients in managing rosacea.

## Data Availability

Not applicable.
